# Multidrug resistant *Pseudomonas aeruginosa* survey in a stream receiving effluents from ineffective wastewater hospital plants

**DOI:** 10.1186/s12866-016-0798-0

**Published:** 2016-08-24

**Authors:** Mary Joyce Targino Lopes Magalhães, Gemilson Pontes, Paula Takita Serra, Antonio Balieiro, Diogo Castro, Fabio Alessandro Pieri, James Lee Crainey, Paulo Afonso Nogueira, Patricia Puccinelli Orlandi

**Affiliations:** 1 Programa de Pós-Graduação Sociedade e Endemias na Amazônia, ILMD, 476, Teresina St, Adrianópolis, 69057-070 Manaus, AM Brazil; 2 Instituto Nacional de Pesquisa da Amazônia – INPA, 2936 André Araújo Av, 69060-001 Manaus, AM Brazil; 3 Instituto de Pesquisa Leônidas e Maria Deane – FIOCRUZ AMAZONIA, 476 Teresina St, Adrianópolis, 69057-070 Manaus, AM Brazil; 4 Departamento Básico – Área de Saúde; Campus Governador Valadares, Universidade Federal de Juiz de Fora, Rua Israel Pinheiro, 2000, Bairro Universitário, Governador Valadares, MG Brazil

**Keywords:** *Pseudomonas aeruginosa*, Amazon, Hospital effluent, Contamination, 16S-rRNA gene, PFGE

## Abstract

**Background:**

Multi-drug resistant forms of *Pseudomonas aeruginosa* (MDRPA) are a major source of nosocomial infections and when discharged into streams and rivers from hospital wastewater treatment plants (HWWTP) they are known to be able to persist for extended periods. In the city of Manaus (Western Brazilian Amazon), the effluent of three HWWTPs feed into the urban Mindu stream which crosses the city from its rainforest source before draining into the Rio Negro. The stream is routinely used by Manaus residents for bathing and cleaning (of clothes as well as domestic utensils) and, during periods of flooding, can contaminate wells used for drinking water.

**Results:**

16S rRNA metagenomic sequence analysis of 293 cloned PCR fragments, detected an abundance of *Pseudomonas aeruginosa* (*P. aeruginosa*) at the stream’s Rio Negro drainage site, but failed to detect it at the stream’s source. An array of antimicrobial resistance profiles and resistance to all 14 tested antimicrobials was detected among *P. aeruginosa* cultures prepared from wastewater samples taken from water entering and being discharged from a Manaus HWWTP. Just one *P. aeruginosa* antimicrobial resistance profile, however, was detected from cultures made from Mindu stream isolates. Comparisons made between *P. aeruginosa* isolates’ genomic DNA restriction enzyme digest fingerprints, failed to determine if any of the *P. aeruginosa* found in the Mindu stream were of HWWTP origin, but suggested that Mindu stream *P. aeruginosa* are from diverse origins. Culturing experiments also showed that *P. aeruginosa* biofilm formation and the extent of biofilm formation produced were both significantly higher in multi drug resistant forms of *P. aeruginosa*.

**Conclusions:**

Our results show that a diverse range of MDRPA are being discharged in an urban stream from a HWWTP in Manaus and that *P. aeruginosa* strains with ampicillin and amikacin can persist well within it.

## Background

*Pseudomonas aeruginosa* (*P. aeruginosa*) is a ubiquitous Gram-negative, opportunistic bacterium that is spread in hospitals on the hands of healthcare workers and by contact with improperly cleaned hospital surfaces and equipment on which it can persist over long periods [1, 2]. *Pseudomonas aeruginosa* can cause, sometimes invasive, infections of burns and surgical wounds that can be life-threating [3, 4]. *Pseudomonas aeruginosa* has intrinsic multidrug resistance and several strains have acquired resistance to a wide variety of antimicrobials, including 3rd and 4th generation cephalosporins and carbapenems [1, 5–8]. Multi drug resistant *Pseudomonas aeruginosa* (MDRPA) are thus a significant and growing source of nosocomial infections that have few treatment options and that are especially problematic for immunocompromised patients and intensive care units (ICU) [9–12].

The dissemination of antimicrobial resistant bacteria from hospital wastewater treatment plants (HWWTP) has been a cause for concern for public health professionals [3, 13–22]. Previous studies have identified MDRPA as the primary pathogen found in the discharge of HWWTP, suggesting that the bacterium could have an important role in the spread of antimicrobial resistance, which is a global and increasing healthcare problem [23]. Consistent with this notion, studies investigating the distribution and persistence of *P. aeruginosa* have shown that the bacterium is widely distributed in the environment and that *P. aeruginosa*, including MDRPA, discharged from HWWTP can persist in the environment over extended periods [24]. The discharge of MDRPA in hospital effluents is thus increasingly being seen as having an important role in the global spread of antimicrobial resistance and is therefore an important public health concern [24, 25].

**Fig. 1:**
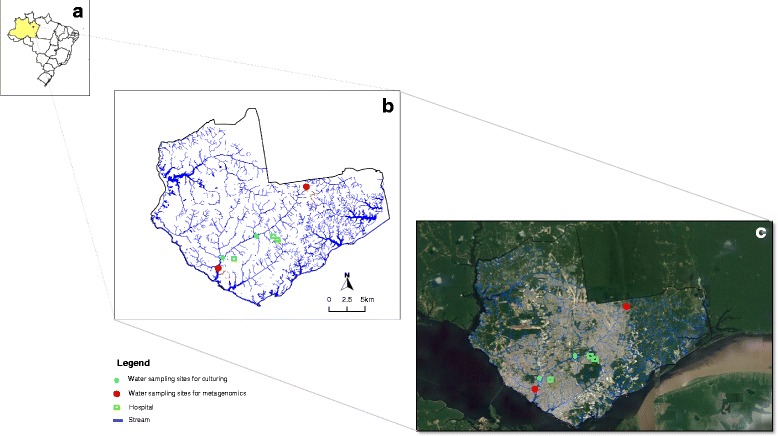
Study setting. (**a**) Manaus city (*red point*), state of Amazonas (*yellow*), Brazil (*outline*). (**b**) Mindu stream crossing Manaus to its Rio Negro drainage site (Outfall). (**c**) Mindu stream flows from its source in the Ducke Forest Reserve and crosses Manaus receiving non-treated wastewater and wastewater from the treatment plants of at least three hospitals. Red points show the sites where surface water samples were collected for the preparation of metagenomic libraries. The blue points show the sites where surface water collections were made from the Mindu stream and green squares show hospitals near to Mindu stream

In this study, we have conducted a preliminary evaluation of the public health threat that MDRPA pose to individuals that live in contact with HWWTP contaminated waters in the city of Manaus (Western region of the Amazon). Using a range of molecular and culturing techniques we have investigated the diversity, persistence and origin of MDRPA in an urban stream in Manaus. The Mindu stream is used by some of Manaus’s most deprived residents for bathing, basic domestic cleaning (of clothes and cooking utensils) and receives discharge waters from three HWWTP as it crosses the city (See Fig. 1). The stream can also contaminate drinking water wells during times of flooding. Our results suggest that HWWTP are polluting the Mindu stream in Manaus with antibiotic resistant *P. aeruginosa* and may be putting residents that live in contact with these contaminated waters at risk to nosocomial infections.

## Results and discussion

### Bacterial diversity at the Mindu stream´s source and drainage sites

As a preliminary and general assessment of the bacterial ecology of the Mindu stream, two bacterial 16S rRNA gene metagenomic libraries were constructed from bacterial concentrates made from the surface water of: (1) a site close to the stream’s source (the S-Library) and (2) a site close to where the stream drains into the Rio Negro (the outfall site, O-Library) (see Fig. 1). A total of 384 16S rRNA clones were randomly chosen for Sanger sequencing from the two libraries, 192 from the S-Library and 192 from the O-Library. Sequence comparisons made with 156 high-quality sequences obtained from the S-Library and reference sequence deposits maintained at the ribosomal Database Project (RDP) and the National Center for Biotechnology Information (NCBI) allowed for 126 (81,0 %) to be confidently classified to the bacterial genus level, including 23 (18,3 %) from the *Pseudomonas* genus. Four of these *Pseudomonas* genus sequences were identical to *P. fluorescens* (17.4 %), two were identical with *P. plecoglossicida* (8.7 %), and one identical sequence match (4.3 %) was found for three other *Pseudomonas* species: *P. putida, P. segetis* and *P. azotoformans*. No *P. aeruginosa* were detected from our S-Library sequencing.

**Fig. 2:**
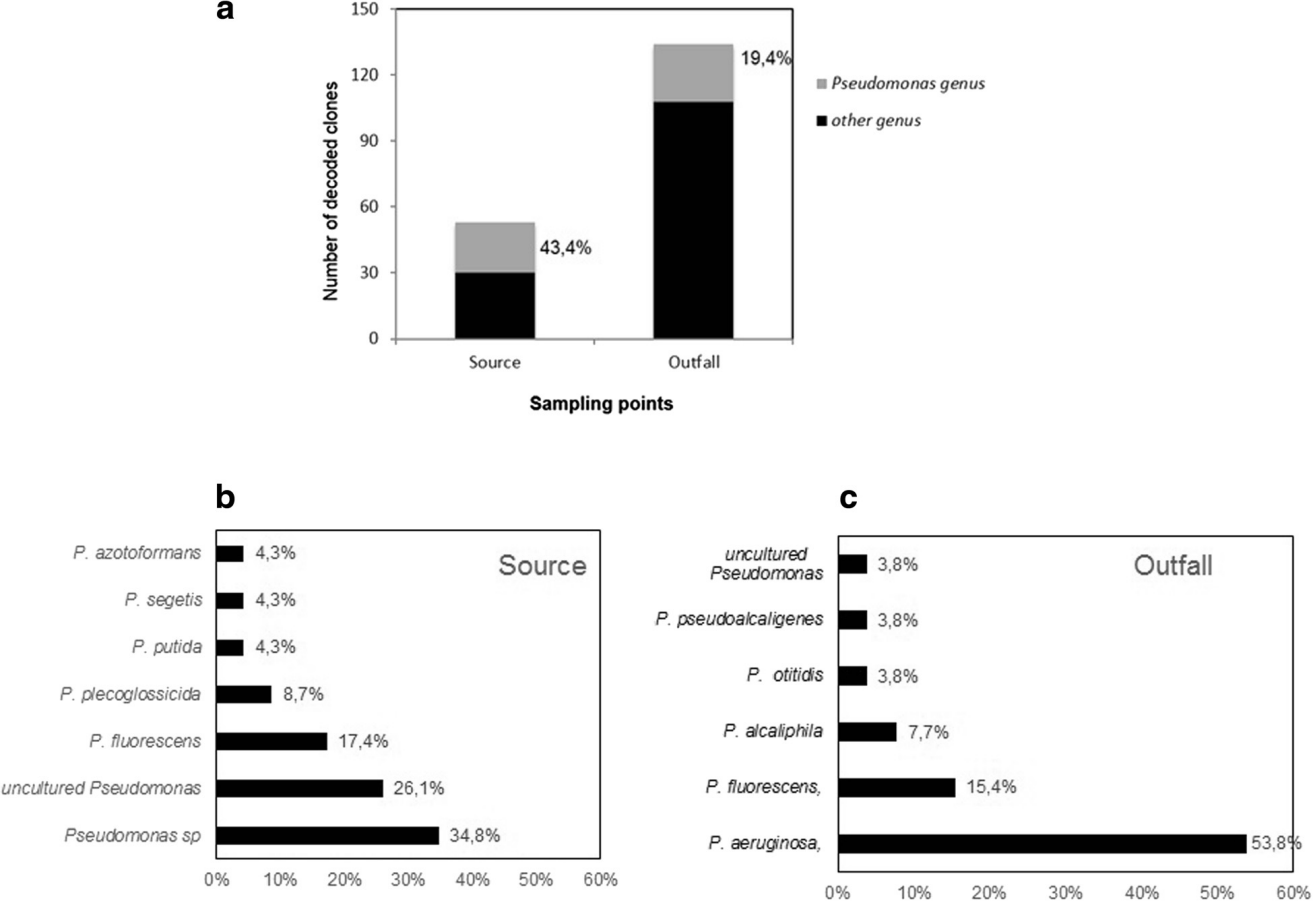
Proportions of *Pseudomonas* genus and species recovered by metagenomic analysis from Source and Outfall of the Mindu stream: **a** proportion of *Pseudomonas* genus **b** proportion of *Pseudomonas* species isolated from the source **c** proportion of *Pseudomonas* species isolated at Outfall

A total of 167 high-quality 16S rRNA gene sequences were obtained from the 192 O-Library clones that were randomly selected for sequencing. Of these high-quality sequences, database similarity matches allowed 134 sequences to be confidently classified to bacterial genus. A very similar proportion of the O-library, 19.6 % (26) as was observed at the S-library (see Fig. 2), were classified by database similarity comparisons as belonging to the *Pseudomonas* genus. However, in contrast to the S-library, a high proportion of the *Pseudomonas* sequences from the O-Library, 14 (53.8 %) were identified as *P. aeruginosa*. Four of the O-Library sequences (15.4 %) were classified as *P. fluorescens* and two (7.7 %) were classified as *P. alcaliphila. Pseudomonas otitidis* and *P. pseudoalcali* sequences were also identified as occurring in the O-library. Hospital effluents are a major source of environmental contamination and contribute substantially to changes in the *Pseudomonas* communities. Our metagenomic data is therefore in agreement with previous studies that have shown that hospital wastewater discharges can change the structure and composition of the bacterial communities that they are discharged into [16, 26–30].

### Antimicrobial resistance profiles of *P. aeruginosa* from the Mindu stream and a Manaus HWWTP

**Fig. 3:**
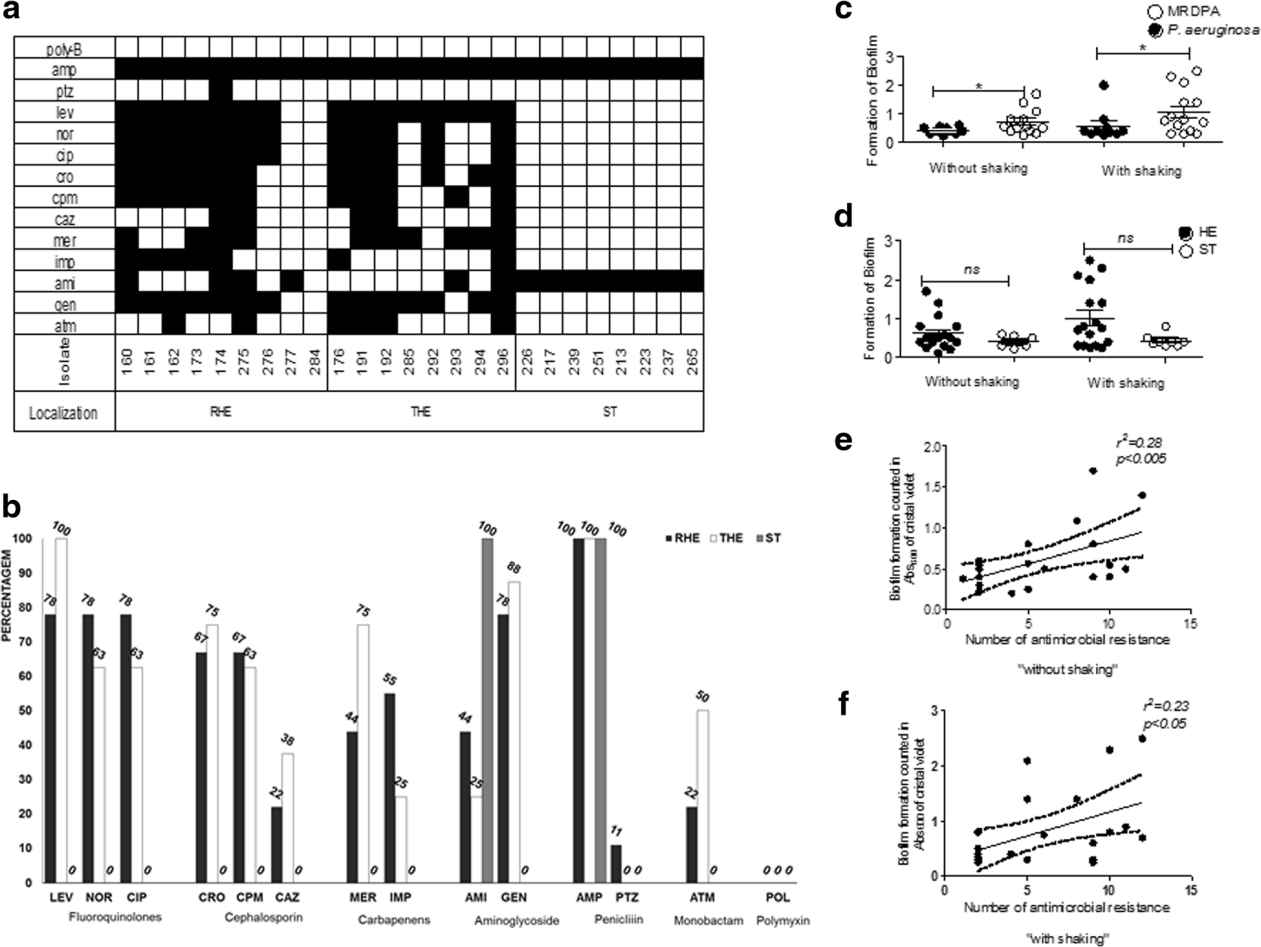
Antimicrobial resistant and biofilm formation analysis. Diagram showing (**a**) the antimicrobial resistance profiles of 25 *P. aeruginosa* isolates obtained from water samples tested in this study. Isolates are labelled with either RHE, THE or ST to indicate their origin: RHE for raw hospital effluent, THE for treated hospital effluent and ST for stream sample. The susceptibility testing was carried out according to the protocol of the Clinical Laboratory Standards Institute (CLSI, 2012) using 14 different antimicrobials: Levofloxacin (LEV); Norfloxacin (NOR); Ciprofloxacin (CIP); Ceftriaxon (CRO); Cefepime (CPM); Ceftazidima (CAZ); Meropenem (MER); Imipenem (IMP); Amicacina (AMI); Gentamicina (GEN); Ampicilin (AMP); Piperaciline-Tazobactam (PTZ); Aztreonam monobactan (ATM); and Polimixina B-I (POL). *Black-squares* are used to indicate resistance and *white-squares* are used to indicate susceptibility. **b** Frequencies of antimicrobial resistant phenotypes were calculated as percentages of antimicrobial resistance among isolates of same origin. **c**-**d** Comparison of biofilm formation in LB broth with or without shaking. Biofilm production expressed as absorbance measurements taken at 600 nm. Comparison between MDRPA versus *P. aeruginosa* (**c**); Comparison between HE versus ST isolates (**d**); **e**-**f** Linear regression analysis showing that the number of antimicrobials a MDRPA is resistant to correlates with the amount of biofilm it produces independently of shaking

A total 25 *P. aeruginosa* isolates were tested for drug susceptibility with a panel of 14 antimicrobials (Fig. 3a and b). Nine *P. aeruginosa* cultures were prepared from bacteria obtained from the raw hospital effluent (RHE) that is fed into the HWWTP for processing and eight were prepared from treated hospital water effluent (THE) samples, which are usually discharged into the Mindu stream. A further eight *P. aeruginosa* cultures were prepared from Mindu stream surface water samples which were taken from two locations, upstream and downstream of the treatment site (Fig. 1). As can be seen in Fig. 3a, a total of 15 widely variant antimicrobial resistance profiles were found among the HWWTP *P. aeruginosa* isolates. Overall the isolates from the HWWTP can be seen to display resistance to between one and 12 antimicrobials, with the RHE displaying resistance to an average of 7.4 antimicrobials and those from the THE displaying resistance to an average of 7.6. In stark contrast to what was observed with the *P. aeruginosa* isolates from the HWWTP, only one resistance profile was detected from the Mindu stream isolates: dual resistance to ampicillin and amikacin. Our results show that *P. aeruginosa* with antimicrobial profiles matching some of the antimicrobial resistant *P. aeruginosa* discharged in the THE are able to persist in the Mindu stream and thus are consistent with what would be expected from the observations of others [23, 31, 32]. Our results thus suggest that our studied HWWTP could be polluting the Mindu stream and could be a public health risk to Manaus residents that live in contact with this stream.

### Genetic characterization of Mindu stream and HWWTP *P. aeruginosa*

**Fig. 4:**
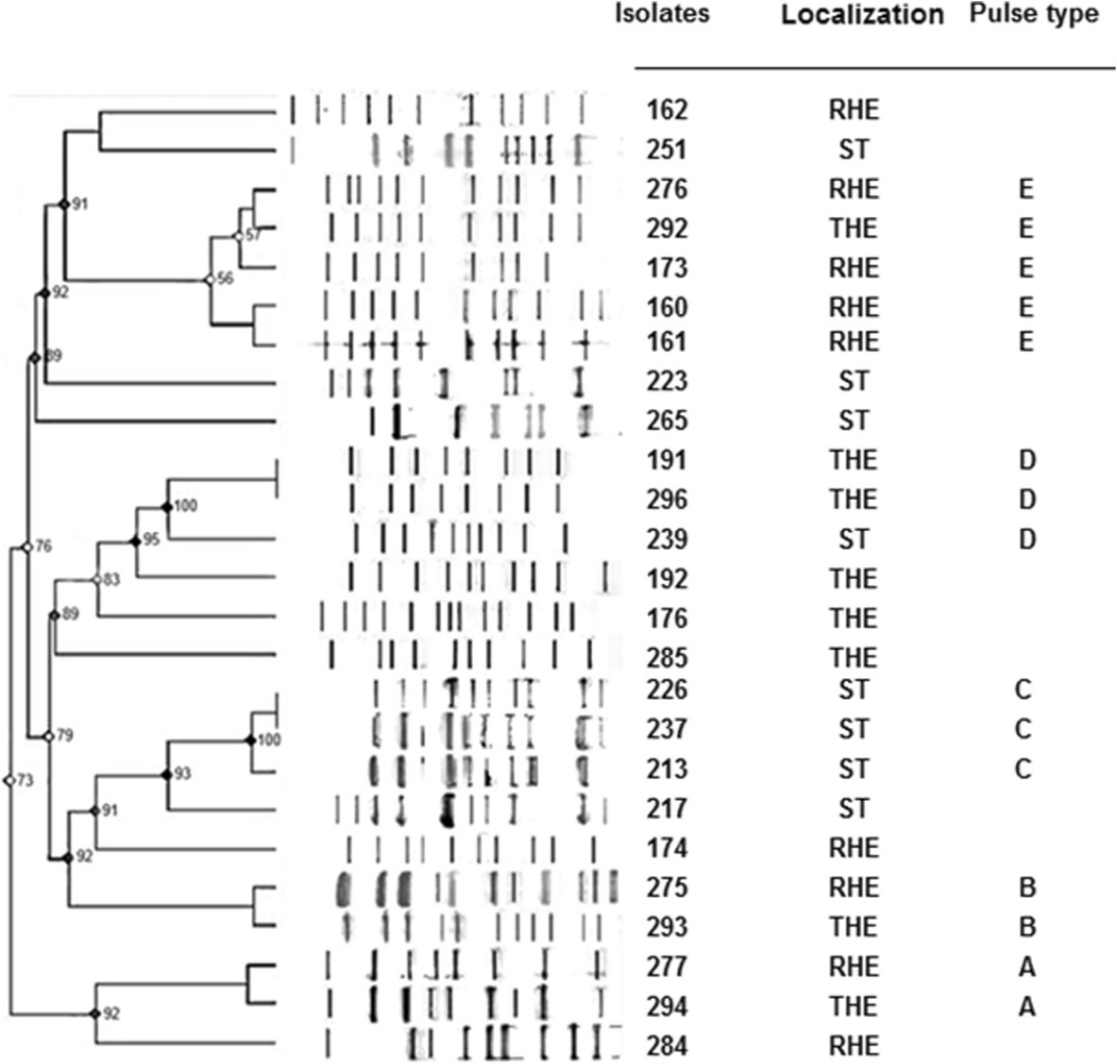
Pulsed-field gel electrophoresis dendogram of 25 isolates of *P. aeruginosa* obtained from hospital effluent (HE) and Mindu stream (ST) water samples (Manaus-AM, Brazil). PFGE profiles were compared and aligned by BioNumerics software. The pulse-types were not classified

Figure 4 shows pulse field gel electrophoresis (PFGE) fingerprints generated for each of the 25 *P. aeruginosa* isolates used in the antimicrobial testing (Fig. 3). These fingerprints were generated by running genomic DNA *XbaI* restriction enzyme digestion products on a PFGE gel. Although clear similarity between these fingerprints are observable between the isolates taken from the HWWTP, there is also clear evidence of high levels of genetic diversity among the RHE and THE bacterial isolates as well as those obtained from the Mindu stream. This approach did not, however, help to resolve the precise origin of antimicrobial resistant *P. aeruginosa* isolates from the Mindu, but did show that, despite the fact they all have identical antimicrobial resistance profiles, they are not all closely related to one another. These results thus suggest that the *P. aeruginosa* of the Mindu stream are most probably derived from multiple origins which adds weight to the notion that the *P. aeruginosa* with resistance to ampicillin and amikacin are at a selective advantage in this stream [23, 31, 32].

### The biofilm formation of Mindu stream MDRPA

All 25 above mentioned *P. aeruginosa* isolates where also assessed for their ability to form biofilms under shaking and non-shaking growth conditions. Biofilm formation was observed to be significantly higher with MDRPA than non-multi-resistant *P. aeruginosa* (p <0.05) while no differences were seen when the isolates were classified based on their origin (Fig. 3c-d). These data indicate thus that biofilm formation is a characteristic of MDRPA. Consistent with this notion, we also observed a positive association between the number of antimicrobials a *P. aeruginosa* isolate was resistant to (i.e., the extent of an isolate’s multi-drug resistance) and the extent of biofilm it formed. Our results are thus consistent with other studies that have suggested that multi-drug resistance is associated with biofilm formation [12, 33–36].

## Conclusions

A HWWTP in Manaus is discharging MDRPA into the urban Mindu stream, where antimicrobial resistant *P. aeruginosa* may be at a selective advantage and are certainly able to persist. Many of Manaus’s most vulnerable residents that live in contact the Mindu stream are being exposed to antimicrobial resistant *P. aeruginosa* and any and all health risks posed by such bacteria. More research is required to determine the extent to which HWWTPs are contributing to the occurrence of antimicrobial resistant *P. aeruginosa* in the Mindu stream and to determine the public health importance of such bacteria.

## Methods

### Waste water collections

**Fig. 5:**
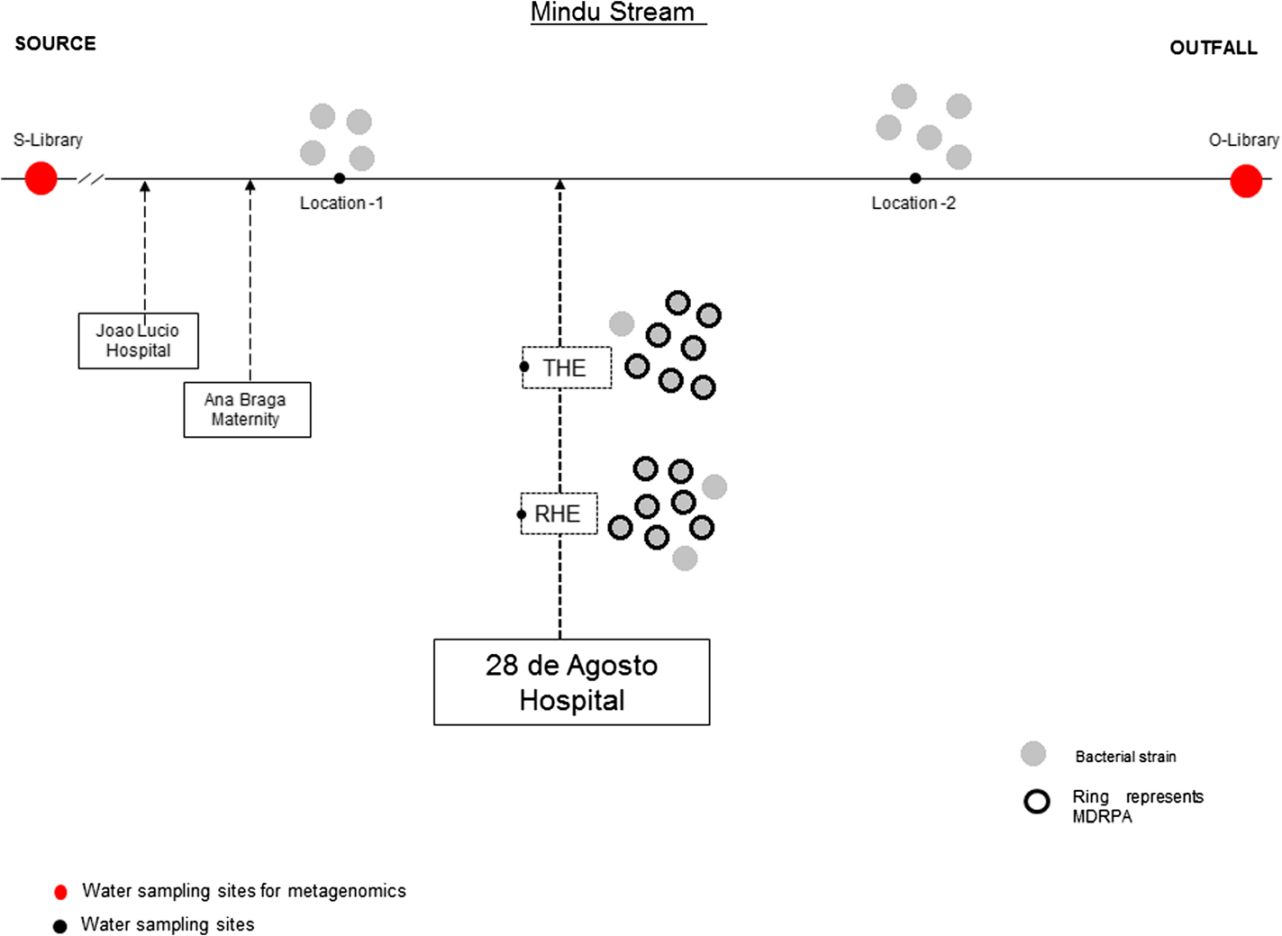
A schematic diagram showing a summary of the results obtained for this study. Abbreviations used in this diagram are the same as those used in other figures

Figure 1 shows a map of the Brazilian city of Manaus, Amazonas state, with the key study localities indicated. Waste water collections from the Hospital Pronto Socorro 28 de Agosto HWWTP were made twice: once in January 2012, and once in July 2012. Surface water samples from the Mindu stream were collected in July 2012 at locations downstream of where the HWWTPs of Manaus discharge their waste water (as indicated in Figs. 1 and 5). Two samples of HWWTP were collected in raw hospital effluent (RHE) and outflow-treated hospital effluent (THE). All water samples were 250 mL in volume and transported refrigerated for laboratory processing. Samples were processed within six hours of collection at Leonidas and Maria Deane Research Institute, located in Manaus. All samples were filtrated with hydrophilic filters with pore sizes of 0.8 μm (Durapore) and 0.22 μm (Sterifil) (Millipore Corp., Bedford, Mass.) using a vacuum pump to concentrate samples. The bacteria collected on these membranes were used to prepare metagenomic libraries and for *P. aeruginosa* isolation. *P. aeruginosa* isolate names reflect when they were collected: samples collected in January all start with the number 1; those collected in July start with the number 2.

### Surface water 16S rRNA gene Metagenomic library preparation

Two metagenomic libraries were made by extracting DNA from bacteria collected on membranes prepared from waste water samples collected at the Mindu stream’s source and outfall sites (see Fig. 1). DNA was extracted using the phenol-chloroform method, before being re-suspended in 40 μL of TEN buffer (100 mM Tris-HCl pH 8.5, 100 mM EDTA, 3 M NaCl) [37]. Bacterial 16S rDNA amplicons of ≈ 600 bp were ampified from this extracted DNA using universal 16S rRNA gene-specific primers: forward 5’ CCTACGGGAGGCAGCAG and reverse 5´ CCGTCAATTCCTTTGAGTTT 3´ [30]. The amplicons obtained from each site were ligated into the pGEM plasmid vector (pGEM®-T Easy, Promega) and used to transform into *E. coli* TOP10 by electroporation. A total of 383 transformed colonies had their 16S rDNA cloned fragments amplified and directly Sanger sequenced using the same primers that were used to amplify them, following an approach similar to one described previously [38]. Generated 16S metagenomic sequences were edited and then taxonomically assigned to genus using both the Bayesian rRNA classifier software from the “Ribosomal Database Project II” (RDP) database [39] and Basic Location Alignment Search Tool (BLAST) software from the National Center of Biotechnology Information (NCBI).

### *Pseudomonas aeruginosa* isolation and antimicrobial susceptibility testing from hospital effluents

Membranes prepared from eight Mindu stream and 17 HWWTP water samples were incubated in 5 mL of LB broth at 37 °C before being plated on to a *Pseudomonas* Isolation Agar (PIA). The 25 *P. aeruginosa* isolates were identified by Gram staining and conventional biochemical testing. In brief, using cytochrome oxidase oxidation and fermentation of glucose and lactose; growth in LB Broth at 42 °C; motility; production of H2S; nitrate reduction and subsequent plating onto *Pseudomonas* Isolation Agar (PIA) for pioverdin production. Antimicrobial susceptibility testing was performed by the agar disk diffusion method against antipseudomonal agents according to Clinical and Laboratory Standards Institute Guidelines, 2012. The following antimicrobials were tested: β-lactamics, including 100ug/10 μg Piperaciline-Tazobactam (PTZ); 10 μg Ampicilin (AMP); 30 μg Aztreonam monobactan (ATM); fluoroquinolones, including 10 μg Norfloxacino (NOR), 5 μg Levofloxacino (LEV), and 5 μg Ciprofloxacin (CIP); 3rd generation cephalosporins, including 30 μg Ceftriaxon (CRO), 30 μg Ceftazidima (CAZ); one 4th generation cephalosporin, 30 μg Cephepime (CPM); aminoglycosides, including 30 μg Amicacin (AMI) and 10 μg Gentamicin (GEN); carbapenens, including 10 μg Imipenem (IMP) and 10 μg Meropenem (MER); and one polymixin B-I 300 U (POL). Isolates resistant to at least three classes of antimicrobials were defined as MDRPA.

### Biofilm formation

Biofilm formation was tested in 15 ml polystyrene tubes following the general approach described by [40]. Biofilm formation was assessed culturing *P. aeruginosa* isolates in 2 ml of LB broth with or without shaking at 37 °C for 24 h. After this, cultures were removed and the adherence pellicle formation was stained with crystal violet for 30 min. After washing in saline phosphate buffer (pH 7.0), the crystals were dissolved in ethanol, and biofilm formation was calculated using optical spectrometry and 600 nm absorbance measurements.

### Pulsed-field gel electrophoresis (PFGE)

Bacterial genomic DNA from the 25 *P. aeruginosa* isolates was fingerprinted by pulsed-field gel electrophoresis (PFGE) following *XbaI* restriction enzyme digestion [41]. PFGE was ran for 20 h and carried-out using the CHEF-DR III system (Bio-Rad, Melville, NY, USA) and a 50,000-bp λDNA PFGE molecular weight marker (Sigma-Aldrich, St. Louis, MO, USA). The DNA fragments were stained by immersing the gels in 400 ml of 0.5 % TBE buffer containing 100 μl of an ethidium bromide stock solution (10 mg/ml) for 30 min. The gels were photographed, and profiles visualized using the GelDoc® (Bio-Rad, Hercules, CA, USA) photo documentation system. The profiles were analysed and compared with BioNumerics software, version 6.5, using the Dice Coefficient and UPGMA comparison settings with tolerance set to 1 %.

### Statistical analysis and data presentation

Data were analysed with GraphPad Prism software version 5. A non-parametric Mann Whitney *t*-Test was used to compare biofilm formation between MDRPA versus *P. aeruginosa* and between HE versus ST isolates. Linear regression analysis was used to test for a correlation between the level of biofilm formation (measured by absorbance at 600 nm) and the amount of antimicrobial resistance that a given *P. aeruginosa* isolate possessed. The results were considered statistically significant when *p* <0.05.

## Abbreviations

HWWTP: Hospital wastewater treatment plants
MDRPA: Multi-drug resistant forms of *Pseudomonas aeruginosa*
O-Library: Outfall library
RSE: Raw sewage effluent
S-Library: Source library
THE: Treated hospital effluent.
